# Hybrid multi-chip assembly of optical communication engines by in situ 3D nano-lithography

**DOI:** 10.1038/s41377-020-0272-5

**Published:** 2020-04-27

**Authors:** Matthias Blaicher, Muhammad Rodlin Billah, Juned Kemal, Tobias Hoose, Pablo Marin-Palomo, Andreas Hofmann, Yasar Kutuvantavida, Clemens Kieninger, Philipp-Immanuel Dietrich, Matthias Lauermann, Stefan Wolf, Ute Troppenz, Martin Moehrle, Florian Merget, Sebastian Skacel, Jeremy Witzens, Sebastian Randel, Wolfgang Freude, Christian Koos

**Affiliations:** 1 Institute of Photonics and Quantum Electronics (IPQ), Karlsruhe Institute of Technology (KIT), Engesserstraße 5, 76131 Karlsruhe, Germany; 2 Institute for Microstructure Technology (IMT), KIT, Hermann-von-Helmholtz-Platz 1, 76344 Eggenstein-Leopoldshafen, Germany; 3 Institute for Automation and Applied Informatics (IAI), KIT, Hermann-von-Helmholtz-Platz 1, 76344 Eggenstein-Leopoldshafen, Germany; 4 Vanguard Automation GmbH, Gablonzer Straße 10, 76185 Karlsruhe, Germany; 5 Fraunhofer Institute for Telecommunications, Heinrich Hertz Institute (HHI), Einsteinufer 37, 10587 Berlin, Germany; 6 Institute of Integrated Photonics (IPH), RWTH Aachen, Sommerfeldstraße 18/24, 52074 Aachen, Germany

**Keywords:** Lithography, Integrated optics, Fibre optics and optical communications

## Abstract

Three-dimensional (3D) nano-printing of freeform optical waveguides, also referred to as photonic wire bonding, allows for efficient coupling between photonic chips and can greatly simplify optical system assembly. As a key advantage, the shape and the trajectory of photonic wire bonds can be adapted to the mode-field profiles and the positions of the chips, thereby offering an attractive alternative to conventional optical assembly techniques that rely on technically complex and costly high-precision alignment. However, while the fundamental advantages of the photonic wire bonding concept have been shown in proof-of-concept experiments, it has so far been unclear whether the technique can also be leveraged for practically relevant use cases with stringent reproducibility and reliability requirements. In this paper, we demonstrate optical communication engines that rely on photonic wire bonding for connecting arrays of silicon photonic modulators to InP lasers and single-mode fibres. In a first experiment, we show an eight-channel transmitter offering an aggregate line rate of 448 Gbit/s by low-complexity intensity modulation. A second experiment is dedicated to a four-channel coherent transmitter, operating at a net data rate of 732.7 Gbit/s – a record for coherent silicon photonic transmitters with co-packaged lasers. Using dedicated test chips, we further demonstrate automated mass production of photonic wire bonds with insertion losses of (0.7 ± 0.15) dB, and we show their resilience in environmental-stability tests and at high optical power. These results might form the basis for simplified assembly of advanced photonic multi-chip systems that combine the distinct advantages of different integration platforms.

## Optics: 3D nano-lithography greatly simplifies photonic multi-chip assembly

Scientists have demonstrated photonic multi-chip modules that rely on 3D-printed waveguides for connecting photonic chips. Current integrated optical systems are often assembled from discrete dies through high-precision alignment techniques, which leads to high fabrication costs and often limits the application range. A team of German researchers led by Christian Koos from Karlsruhe Institute of Technology (KIT) has now demonstrated optical communication engines that exploit 3D-printed freeform waveguides, so-called photonic wire bonds, as chip-chip connections. The photonic wire bonds are fabricated in-situ onto the chips by advanced 3D lithography and can efficiently connect a wide variety of photonic integration platforms. By greatly simplifying the assembly of advanced photonic multi-chip modules, the technique has the potential to transform a variety of applications, ranging from high-speed communications and ultra-fast signal processing to optical sensing and quantum information processing.

## Introduction

Photonic integration is a key technology that has the potential to transform a wide variety of applications, ranging from high-speed communications^1^ and ultra-fast signal processing^2,3^ to optical metrology and sensing^4^ and further to quantum information processing^5,6^. At present, most commercial products in the field of integrated optics still rely on discrete assemblies of photonic chips and require additional coupling elements, such as on-chip mode-field adapters^7^ or rather bulky micro-lenses and redirecting mirrors^8^. Assembling such systems from discrete chips allows combining the complementary advantages of different photonic integration platforms but requires technically complex active alignment techniques, which rely on continuous monitoring of the coupling efficiency while positioning and mounting the devices^9^. These techniques are hence characterized by high cost and low throughput, thereby nullifying most of the inherent advantages offered by wafer-scale mass production of photonic integrated circuits (PIC)^10^. These challenges may be overcome by monolithic integration^11^, leading to PIC that combine all elements on a common substrate. However, while monolithic integration minimizes the number of costly inter-chip connections and thus provides utmost scalability, the functionality and performance of the resulting PIC are often limited by the optical properties of the underlying material system. A prime example in this context is the silicon photonic (SiP) platform^12^, which exploits advanced CMOS processing to provide unparalleled scalability but suffers from an indirect bandgap that inhibits efficient light emission as well as from a lack of second-order nonlinearities, which limits the performance of its electro-optic modulators. Moreover, monolithic co-integration of multiple devices on a single die requires complex fabrication processes and thus crucially relies on tight process control to achieve acceptable yield levels. This leads to significant technological overhead, which, in many cases, is in conflict with the heterogeneous and highly fragmented application space of photonic integrated circuits^13^.

In this paper, we show that the performance and flexibility of conventional discrete-die systems can be combined with the compactness and scalability of monolithically integrated circuits by exploiting advanced additive nanofabrication techniques. Our approach relies on direct-write two-photon lithography^14^ for in situ fabrication of three-dimensional (3D) freeform polymer waveguides between coarsely pre-positioned photonic devices. This technique, also referred to as photonic wire bonding^15–17^, does not require active alignment and allows for highly efficient optical coupling between a broad range of waveguide types with vastly different mode-field profiles in a fully automated process. Building upon previous proof-of-concept experiments that show the basic applicability of the approach to chip–chip^17^ and fibre-chip^15^ interfaces, we systematically investigate and demonstrate the reproducibility, reliability, and scalability of the concept. In our experiments, we fabricated 100 densely spaced PWBs with an average total insertion loss of 0.7 dB and a standard deviation of 0.15 dB, and we demonstrate their reliability in temperature cycling and damp-heat tests. To prove the practical applicability of our approach, we further realize two different optical transmitter engines that combine arrays of direct-bandgap InP-based light sources with SiP modulators. As a first demonstration, we show an eight-channel intensity modulation/direct detection (IM/DD) transmitter engine that comprises an individual InP laser, a SiP modulator and a fibre pigtail for each channel. This hybrid multi-chip module allows transmission of an aggregate line rate of 448 Gbit/s over a 10 km-long unamplified optical link^18^. As a second demonstration, we realize a four-channel coherent transmitter module utilizing highly efficient silicon-organic hybrid (SOH) modulators to overcome the intrinsic lack of second-order nonlinearities of the SiP integration platform^19^. Transmitting 56 GBd QPSK and 16QAM signals at a line rate of 784 Gbit/s over a distance of 75 km, this module provides the highest data rate demonstrated by a SiP transmitter module with co-integrated lasers to date. We believe that these experiments mark an important step towards exploiting the flexibility and design freedom offered by additive 3D nanofabrication in the field of photonic integration.

## Results and discussion

### Hybrid multi-chip integration by photonic wire bonding

**Fig. 1:**
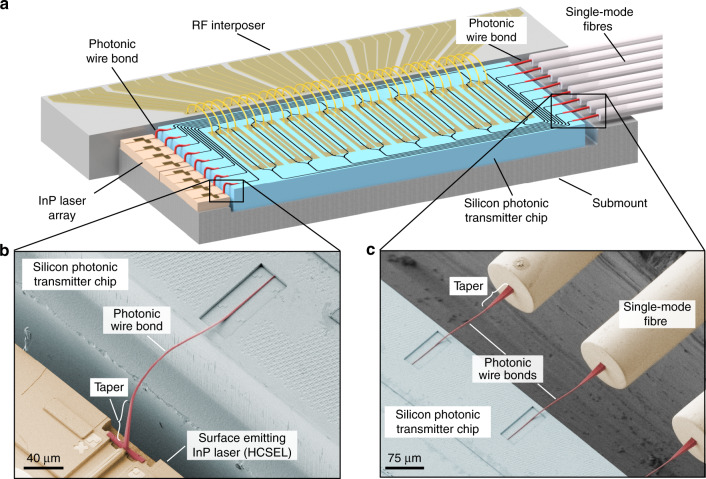
Concept and implementation of hybrid multi-chip modules (MCMs) by 3D nano-printing of photonic wire bonds (PWBs). **a** Illustration of an eight-channel transmitter, realized as a hybrid MCM comprising 3D-printed PWBs shown in red. PWBs allow efficiently connections between photonic integrated circuits (PICs) that are realized on different integration platforms, thereby combining the complementary strengths of the underlying material system. The illustrated transmitter combines efficient InP lasers with electro-optic modulators on a silicon photonic chip. The modulator array is electrically driven via an RF fan-in and connected to an array of single-mode fibres. **b** Interface between an InP laser chip and the silicon photonic transmitter chip. The light source is realized as a horizontal cavity surface emitting laser (HCSEL), consisting of a waveguide-based optical cavity in the substrate plane and an etched 45° mirror that redirects the light towards the substrate-normal direction^17^. **c** Fibre-to-chip interface. For efficient coupling to the large mode-field of the SMF, the PWBs are designed to have a larger cross section towards the fibre facet. The 3D freeform trajectory of the PWBs is adapted to the exact position of the corresponding interfaces and thereby replaces high-precision active alignment of the chips

The concept of a hybrid multi-chip module (MCM) using photonic wire bonds is illustrated in Fig. 1a using an optical transmitter engine as an example. The module combines multiple photonic dies based on different material systems, such as indium phosphide (InP) or silicon-on-insulator (SOI). In a first step, these chips are fixed to a common submount together with an array of single-mode fibres (SMFs). This step does not require any high-precision alignment such that cost-efficient high-throughput pick-and-place equipment with rather coarse positioning tolerances of 10 µm or more can be used. Chip-to-chip and fibre-to-chip connections are then realized by 3D freeform photonic wire bonds, the cross section and trajectory of which can be flexibly adapted to the mode profile and location of the respective optical interfaces. For fabrication of the PWBs, on-chip alignment markers are detected by high-resolution 3D imaging and computer vision techniques to extract the exact position and orientation of the various optical components and their interfaces. This information is used for designing the PWB trajectories, thus replacing costly chip alignment by the intrinsic sub-100 nm accuracy of the lithography and imaging system. The PWBs are then fabricated by two-photon lithography, offering sub-µm resolution in all spatial directions; see the Methods section for details of the fabrication process. In our experiments, the PWBs feature a core refractive index of *n*_core_ = 1.52 and a waveguide cross section of 2.0 × 1.6 µm^2^ such that the structure is mechanically stable and still small enough for single-mode operation when embedded into a protective low-index cladding material (*n*_clad_ = 1.36). This configuration allows for sharp bends with radii down to 35 µm, thus enabling flexible waveguide routing in compact multi-chip assemblies^15,17^. Note that photonic wire bonding allows placing the optical chips side by side, which permits efficient thermal connection to the submount and the underlying heatsink and thus prevents thermal bottlenecks that may arise in stacked-chip assemblies, e.g., when light sources are mounted on top of thick substrates with low thermal conductivity.

The MCM illustrated in Fig. 1a crucially relies on efficient connections of the SiP chip to both the InP light source and the output transmission fibre, which are shown in more detail in Fig. 1b, c. The light sources are realized as horizontal cavity surface emitting lasers (HCSELs), which comprise an InGaAsP-based distributed-feedback cavity in the substrate plane and an etched 45°-mirror that redirects the light towards the substrate normal^20^. Both ends of the PWB feature mode-field converters for low-loss coupling to the connected devices, Fig. 1b. On the laser side, the PWB cross section is increased to match the larger mode-field diameter (3.5 µm) of the HCSEL^17^. A similar structure is used at the fibre-chip interface, shown in Fig. 1c. Coupling to the SiP chip is accomplished by a double-taper structure that consists of a SiP nanowire embedded in a polymer waveguide; see the Methods section and ref. ^17^ for details. For low-loss coupling to the SMF, the PWB is tapered to a 14 × 14 µm^2^ cross section to match the mode field diameter to that of the fibre. The PWBs shown in Fig. 1c were designed to compensate for a lateral offset of 25 µm between the axes of the optical fibres and the corresponding SiP waveguides. Note that the density of photonic wire bonds along the circumference of the chip can be greatly increased by abandoning the industry-standard pitches of 250 µm that were used for the lasers and the fibres in our experiments. As shown in the following section, PWBs can be realized with pitches of 25 µm, and this could be reduced even further to 10 µm, allowing for 100 PWBs per millimetre of chip edge. When combined with micro-lenses, PWBs can also be used for optical out-of-plane connections to the chip surface^21^.

### Scalability and stability of photonic wire bonds

**Fig. 2:**
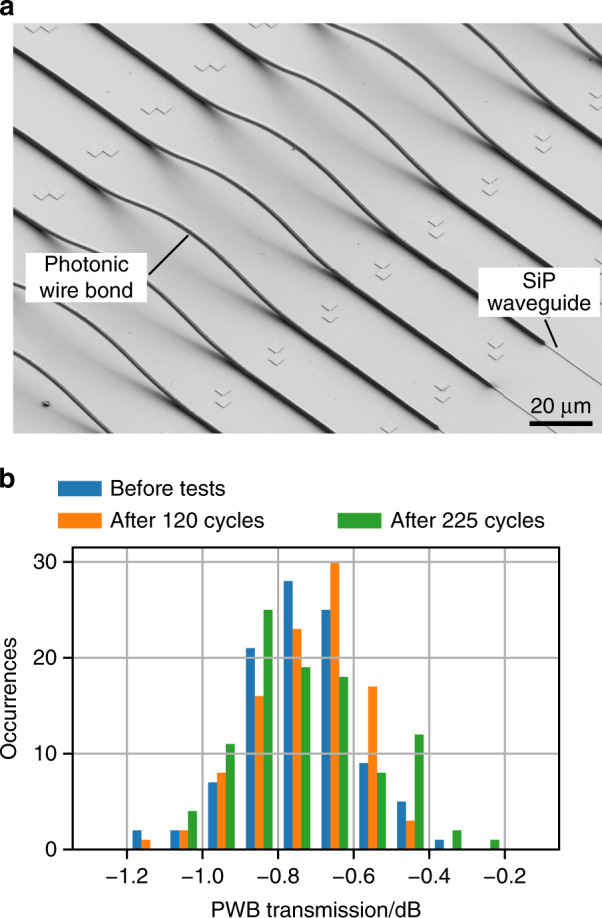
Automated fabrication and environmental stability. **a** Array of densely spaced on-chip PWB test structures. The scanning electron microscope (SEM) image depicts a subset of an array of 100 PWBs realized on a dedicated a silicon photonic (SiP) test chip. The PWB bridges connect tapered ends of SiP strip waveguides, separated by 100 µm. High-resolution 3D imaging in combination with computer vision is used for automated detection of the optical coupling with high precision (better than 100 nm) and enables highly reproducible lithographic definition of the freeform structures. The waveguides are finally embedded into a UV-curable low-index polymer (not shown), which acts as a protective cladding and allows adjustment of the refractive-index contrast. **b** Histogram showing measured insertion losses of 100 on-chip PWB bridges directly after fabrication (blue) as well as after temperature cycling tests, comprising 120 (orange) and 225 (green) cycles. The indicated transmission comprises the propagation loss in the freeform polymer waveguide of the PWBs as well as the overall loss of both double-taper interfaces to the adjacent SiP strip waveguides. After fabrication, the PWB bridges exhibits an average insertion loss of 0.73 dB and a standard deviation of 0.15 dB, and the loss of the worst structure was 1.2 dB. These figures are essentially unaffected by the temperature cycles. The slightly different shapes of the histograms are attributed to the fact that the samples had to be removed from the measurement setup for temperature cycling, leading to small changes in fibre-chip coupling efficiency

In a first experiment, we demonstrate that PWBs can provide low-loss optical connections. To this end, we use dedicated test chips that allow for efficient fabrication and testing of statistically relevant numbers of PWB connections using automated fabrication and characterization tools. These test chips consist of pairs of down-tapered SiP strip waveguides^16^ that can be connected by on-chip PWB bridges; see Fig. 2a and Supplementary Fig. S1. The taper tips having a nominal width of 130 nm are spaced by a 100 µm-wide gap, which emulates a typical configuration of a chip–chip interface and which is bridged by a freeform PWB. The SiP strip waveguides are 500 nm wide and 220 nm high and are equipped with grating couplers for measuring the transmission spectrum in an automated setup. The test chips were fabricated by deep-UV lithography in a standard CMOS line. Each test chip contains 100 test structures along with reference structures that consist of uninterrupted SiP waveguides without PWB bridges or tapers and that allow for separating the PWB loss from the fibre-chip coupling loss; see Supplementary Section S1.

The in situ fabrication of a PWB is fully automated and took ~30 s per connection, where 15 s were spent for interface detection and trajectory routing, and 15 s were used for exposure of the resist. This process can be further accelerated; see the Methods section for details. In the experiment, we fabricated a total of 100 densely spaced PWB bridges with a pitch of 25 µm on a single chip. To extract the insertion loss (IL) of the PWB bridges, we first measure the end-to-end transmission through grating couplers of the connected SiP waveguides at a wavelength of 1550 nm and then compare this result to the transmission of the reference structures. We thus obtain the total loss of the PWB connection, comprising the propagation loss in the freeform polymer waveguide as well as the loss of both double-taper interfaces. To achieve single-mode operation of the PWB bridges and to protect the structures, we locally deposit a polymer cladding (*n*_clad_ = 1.36); see the Methods section for details. The measured ensemble of 100 PWB bridges exhibits an average IL of 0.73 dB and a standard deviation of 0.15 dB, and the loss of the worst structure was 1.2 dB. Comparable results were obtained by repeating the experiment on other test chips. This clearly demonstrates the excellent reproducibility and yield of fully automated photonic wire bonding processes.

To prove the reliability of the structures under technically relevant environmental conditions^22^, the sample is subsequently exposed to multiple temperature cycles, switching between −40 °C and 85 °C. Within the accuracy of our measurements, no performance degradation is found even after 225 cycles; see Fig. 2b. Moreover, the samples do not reveal any signs of degradation, such as delamination of the cladding material; see Supplementary Section S1. These findings confirm results from investigations of earlier samples, which we exposed to 600 temperature cycles as well as to damp-heat at 85 °C and 85% relative humidity for more than 3000 hours, where we did not observe any degradation either. To test the high-power handling capabilities of the PWB structures, we further subject a different sample of PWB bridges to continuous laser radiation at 1550 nm with increasing optical power levels. In all five tested connections, the SiP waveguides were destroyed by non-linear absorption at ~19 dBm of on-chip power before any damage was observed at the PWB bridges; see Supplementary Section S1 for details. From these experiments, we conclude that PWBs lend themselves to automated large-scale packaging of chips using low-loss connections and that the structures perform well in industrially relevant environments and under power levels that are realistically achieved in silicon photonic assemblies.

### Demonstration 1: Eight-channel multi-chip transmitter module for intensity modulation and direct detection

To demonstrate the technical viability of the PWB approach beyond fundamental proof-of-concept demonstrations, we realize a functional photonic multi-chip transmitter (Tx) engine that combines InP-based laser arrays and SiP modulator arrays. In a first demonstration experiment^18^, we implement the eight-channel transmitter (Tx) depicted in the conceptual drawing in Fig. 1, providing line rates of up to 56 Gbit/s per channel. The module is geared towards transmission in data-centre and campus-area networks with maximum distances of up to 10 km using technically simple intensity modulation and direct detection techniques.

**Fig. 3:**
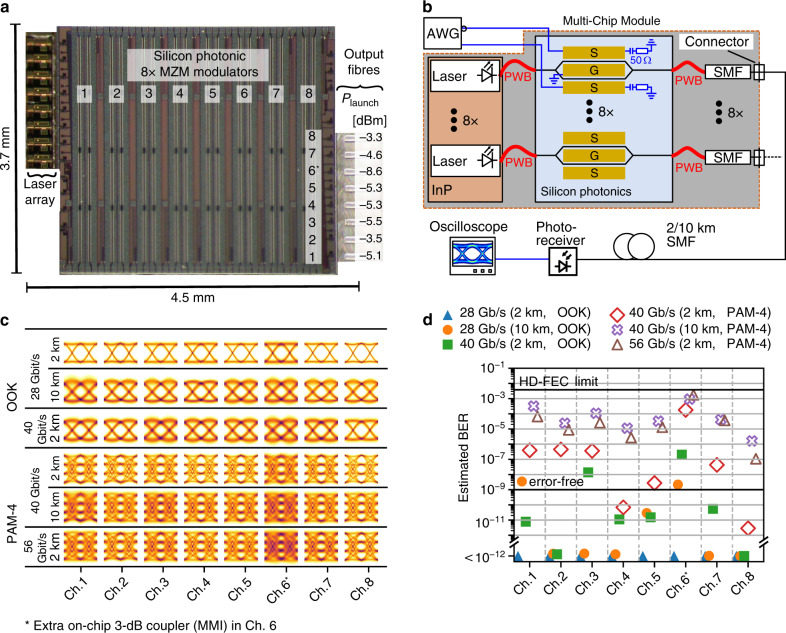
Eight-channel multi-chip transmitter (Tx) module combining InP laser arrays and SiP modulator arrays. The module is geared towards transmission in data-centre and campus-area networks with maximum distances of 10 km, using simple intensity modulation and direct detection techniques. **a** Light-microscope image of the Tx assembly, realized according to the concept shown in Fig. 1a. The array of Mach-Zehnder modulators (MZMs) is connected to an array InP-based HCSEL (“Laser array”) and to an array of single-mode fibres by PWBs (not visible here); see Fig. 1b, c. The launch powers, measured in the single-mode fibre for maximum transmission of the modulators, are sufficient for transmission over distances typical for data centre and campus-area networks, without the need of optical amplifiers. Launch power variations are mainly attributed to non-ideal coupling to and from the SiP chip; see the Methods section for details. Channel 6* contains an additional on-chip 3 dB splitter for testing, which leads to additional loss; see the Methods section. **b** Experimental setup for transmission demonstrations using different modulation formats and distances. An arbitrary-waveform generator (AWG) is used to drive the MZMs. In our demonstration, the modulators are operated sequentially via an RF probe delivering the drive signal at the input and another RF probe to provide a 50 Ω termination at the output. The optical signal is sent through up to 10 km of standard SMF and is detected with a photoreceiver that contains a photodetector along with a high-speed transimpedance amplifier. A real-time oscilloscope is used to capture the electric signals for subsequent offline processing. **c** Eye diagrams for transmission over various distances, with different modulation formats and symbol rates. As expected from the launch powers, Channel 8 shows the widest-open eyes, whereas Channel 6 is distorted by noise. **d** Estimated bit error ratios (BERs) for transmission over various distances, with different modulation formats and symbol rates. For all experiments, the BER stays below the 7% HD-FEC threshold. The aggregate module line rate amounts to a 448 Gbit/s. Results from back-to-back transmission experiments as well as measured BERs can be found in Supplementary Section S2

An optical microscope image of the Tx assembly is shown in Fig. 3a. The assembly contains two arrays of four HCSELs^20^, which are connected via PWBs to an array of travelling-wave depletion-type Mach-Zehnder modulators^23^ (MZMs). A second array of PWBs is used to connect the modulator outputs to an array of eight 30 cm-long SMFs with connectors at their remote ends. Representative images of the PWB structures are shown in Fig. 1b, c. The experimental setup for testing the Tx module is shown in Fig. 3b. The modulators are sequentially driven via microwave probes using a benchtop-type arbitrary-waveform generator (AWG) that provides either two-level on-off-keying (OOK) or four-level pulse amplitude modulated (PAM-4) signals. Pre-emphasis is used to compensate for the frequency response of the AWG and the attached RF components. The modulated optical signals are sent either directly to the receiver (Rx) in a back-to-back (b2b) configuration or through 2 km-long or 10 km-long SMFs. The receiver consists of a photodetector with an integrated transimpedance amplifier connected to a high-speed oscilloscope that records the electric signal for subsequent offline digital signal processing (DSP). Note that in our experiment, we did not use an RF interposer board that would allow for simultaneous operation of all MZMs, and various channels could hence only be tested sequentially. To confirm that simultaneous operation of all devices would lead to similar results, we measured the electrical cross-talk among unterminated MZMs; see Supplementary Section S2.1 for details. More details on the transmission experiment and the data processing can be found in the Methods section.

For both modulation formats, OOK and PAM-4, the eye diagrams of each channel in various combinations of line rates and transmission distances of up to 10 km are shown in Fig. 3c, and the associated estimated bit error ratios (BERs) are depicted in Fig. 3d. All observed BERs are below the 7% hard-decision forward-error correction^24^ (HD-FEC) limit. Note that BER < 10^−5^ cannot be reliably measured within our maximum symbol recording length. We therefore estimate the BER based on the measured variances of a Gaussian probability density function at each symbol level; see Supplementary Information S2.2 for details. For PAM-4, simultaneous operation of all channels would result in an aggregate line rate of 448 Gbit/s and a net data rate of 416 Gbit/s. The concept would hence lend itself to compact high-speed 400 Gbit/s modules as specified in various standards and multi-source agreements^25,26^. For the case of OOK, we demonstrate an aggregate line rate of 320 Gbit/s over a transmission distance of 2 km with an estimated BER < 1.0 × 10^−9^ for all channels. Note that the current demonstration is a proof-of-concept experiment that leaves room for optimization. As an example, the optical launch power levels measured in the single-mode transmission fibres for full transmission of the MZMs vary between −3.3 dBm and −5.5 dBm, disregarding Channel 6, which is subject to an additional on-chip 3 dB tap; see the Methods section for details. From these results, we estimate insertion losses between 3.6 dB and 5.3 dB per PWB. These loss figures are significantly larger than the numbers found in the scalability experiment shown in Fig. 2 or in more recent demonstrations of chip-to-chip connections^17^. This is mainly caused by a non-optimum etching process of the SiP chip, which is required to open the oxide windows for providing access to the tapered sections of the silicon waveguides; see the Methods section. The transmission rate could be further increased by replacing the SiP depletion-type MZMs with faster devices based on organic electro-optic materials, which have been demonstrated to support data rates of more than 100 Gbit/s per wavelength^27^ for OOK and 120 Gbit/s for PAM-4^28^. In our experiment, the emission frequencies of the HCSEL light sources in each array are spaced by 100 GHz. Instead of using eight separate SMFs, a hybrid co-integrated arrayed-waveguide grating (AWG) could extend the functionality of the module to dense WDM transmission through only one SMF. Note that PWBs have been demonstrated^16^ to exhibit broadband transmission between 1300 nm and 1600 nm such that the concept can be readily transferred to other wavelength bands.

### Demonstration 2: Four-channel multi-chip transmitter module for coherent communications

In a second demonstration, we realize and test a hybrid multi-chip transmitter module for coherent communications in metropolitan-area networks and data-centre interconnects. In this module, hybrid multi-chip integration with PWBs is combined with hybrid on-chip integration of electro-optic modulators that combine SiP nanowire waveguides with highly efficient electro-optic materials. This so-called silicon-organic hybrid (SOH)^29^ approach allows overcoming the intrinsic lack of second-order optical nonlinearities in the inversion-symmetric diamond crystal lattice of silicon. SOH devices offer voltage-length products *U*_*π*_*L* of less than^30^ 0.5*V* mm—more than an order of magnitude below that of conventional depletion-type devices^31^—and have been shown to support line rates^27^ of 100 Gbit/s for a simple OOK modulation format and symbol rates of up to 100 GBd for 16-state quadrature amplitude modulation^31^ (16QAM) at fJ/bit energy consumption^32^.

**Fig. 4:**
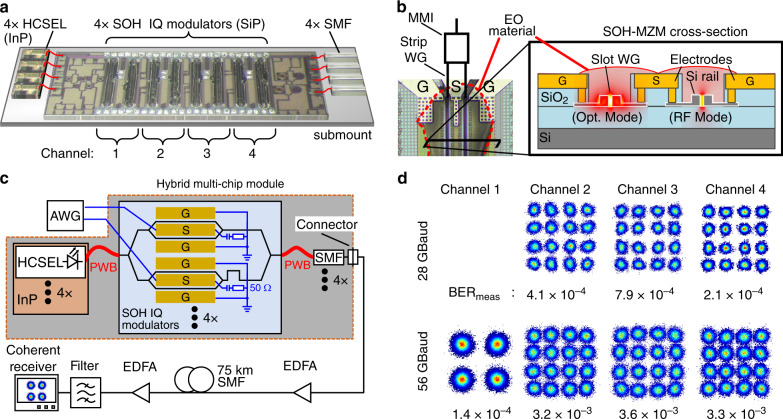
Four-channel coherent transmitter module combining hybrid integration concepts on the chip and package levels. **a** Artist’s impression of the multi-chip-module (MCM) consisting of four InP-based HCSEL light sources, an array of four silicon-organic hybrid (SOH) modulators, and four transmission fibres, all connected by photonic wire bonds (PWBs). The overall footprint of the complete Tx module amounts to 4 × 1.5 mm^2^. **b** Top view and cross section of an SOH Mach-Zehnder modulator (MZM). The organic electro-optic (EO) material (red contour) is micro-dispensed after fabrication of the PWB. The MZM consists of two slot-waveguide (WG) phase modulators, driven in push-pull mode by a single coplanar transmission line in ground-signal-ground (GSG) configuration. Within the slot-waveguide phase shifters, the dominant electrical component of the optical quasi-TE mode exhibits a strong overlap with the electrical RF-mode field, resulting in a high modulation efficiency^32^. **c** Experimental setup. Each HCSEL feeds an IQ modulator. Electric drive signals for the modulators are provided by an arbitrary-waveform generator (AWG). The optical signal is then amplified, sent through 75 km of standard SMF, and detected by a coherent receiver. A real-time oscilloscope captures the signal for subsequent offline processing; see the Methods section for details. **d** Constellation diagrams and associated measured bit error ratios (BERs) for signalling with 16QAM at symbol rates of 28 GBd and 56 GBd. The performance of Channel 1 was impaired by lower launch power such that only QPSK transmission could be used. All BER values stay below the threshold for hard-decision forward-error correction FEC with 7% coding overhead. The aggregate module line rate amounts to 784 Gbit/s

An illustration of the Tx module is shown in Fig. 4a. The module consists of a SiP chip that comprises four SOH IQ modulators along with the associated optical coupling interfaces and RF contact pads. These modulators are again optically fed by an array of InP-based HCSEL light sources that are placed adjacent to the SiP chip on the same submount. At the output, the SOH modulators are connected to an array of four single-mode fibres for transmission over 75 km. As in the first demonstration, the emission frequencies of the HCSEL are spaced by 100 GHz, and instead of using four separate SMFs, hybrid co-integration with an arrayed-waveguide grating could extend the functionality of the module to dense WDM transmission through a single SMF.

The IQ modulators are built from pairs of nested SOH MZMs. A top view and a schematic cross section of one MZM are illustrated in Fig. 4b. Each MZM comprises two SOH phase modulators that are driven in push-pull mode by a single coplanar transmission line in a ground-signal-ground (GSG) configuration. The phase modulators consist of slot waveguides clad with an organic electro-optic (EO) material; see the cross section in the inset of Fig. 4b. The slot-waveguide configuration leads to a strong overlap of the fundamental optical quasi-TE mode with the modulating RF field, which is applied via the Si rails of the slot waveguide and the adjacent conductive Si slabs^29^. In combination with the strong electro-optic activity, this effect results in highly efficient devices with low power consumption. The basic SiP waveguide structures are fabricated by a CMOS process, and the organic EO material is deposited in a post-processing step; see the Methods section for details. The π-voltage-length product measured for this module amounts to $U_\pi L = 1.3\,V\,{\mathrm{mm}}$. This is larger than the values published in earlier work^30^ since the EO material (SEO100) was chosen for good thermal stability for operation at 80 °C rather than to maximize efficiency.

In our demonstration experiment, the PWBs were operated contrary to their design without protective cladding to avoid the risk of covering the electrical on-chip contact pads. This leads to rather high insertion PWB losses, both for the laser and the fibre interface; see Methods for details. Regarding the launch power, Channels 2, 3, and 4 feature similar performance with power levels of up to −11.6 dBm. In Channel 1, the PWB on the HCSEL side is impaired by a dirt particle that permanently attached to the PWB during the fabrication process, which is partially accomplished outside in a normal laboratory environment outside a clean room. This leads to a reduced launch power of only −19.9 dBm and hence to lower transmission performance. Such problems can be avoided by performing all fabrication steps in a clean-room environment as done for samples used in the reliability tests; see Fig. 2. On these chips, the PWBs were directly encapsulated after fabrication and did not show any dirt-related defects. We believe that once the whole transmitter module is assembled and encapsulated under clean-room conditions, contamination by dirt particles will not be an issue. Nevertheless, we demonstrate the functionality of the module in a coherent transmission experiment. To this end, we use the setup shown in Fig. 4c. An AWG is used to drive the IQ modulators through two GSG probes. For each channel, the modulated optical carrier is sent through a 75 km-long SMF. At the receiver, the signal is boosted by an optical pre-amplifier and then coherently detected by an optical modulation analyser and an external-cavity laser (ECL) serving as a local oscillator. Details of the transmission experiment can be found in the Methods section. A summary of the recorded constellation diagrams and the associated BER is shown in Fig. 4d. Channel 4 has the cleanest constellation diagrams, whereas Channel 1 supports QPSK only due to its lower launch power. All BERs are directly measured and fall below the limit of 3.8 × 10^−3^ for second-generation hard-decision forward-error correction (FEC) with 7% overhead^33^. Details on signal processing and results from back-to-back measurements can be found in the Methods section and Supplementary Section S3. The coherent transmission experiments lead to an aggregate line rate of 784 Gbit/s and a net data rate of 732.7 Gbit/s. To the best of our knowledge, this corresponds to the highest values so far demonstrated with a SiP transmitter module having hybrid integrated lasers. In our transmission experiments, we did not observe any distortions due to frequency fluctuations or excessive phase noise of the optical carriers, although our assembly did not contain any optical isolator between the SiP modulator chip and the InP HCSEL. This is in accordance with previous demonstrations, where the optical linewidths of HCSEL with low-loss PWB connections to SiP chips were investigated^17^. The footprint of the four-channel coherent Tx module is below 4 × 1.5 mm, including the SOH chip, the InP light sources and the PWB to the transmission fibre. This corresponds to an on-chip area of only 1.5 mm^2^ per coherent transmitter and is comparable to that of monolithically integrated InP-based transmitters^34,35^.

## Summary and outlook

We have shown that in situ 3D nanofabrication of photonic wire bonds (PWB) overcomes the limitations of current hybrid photonic integration approaches, namely, the high placement accuracy of elements required during assembly and the necessity to match vastly different mode field sizes. In a first experiment, we used SiP test structures to demonstrate highly efficient and reproducible coupling losses of (0.73 ± 0.15) dB measured from 100 PWBs. The PWBs were fabricated in a fully automated process and tested under technically relevant environmental conditions without failures or degradation. We further demonstrate the viability of PWBs by realizing two different hybrid multi-chip transmitter engines. A first module is built from eight independent InP lasers connected to arrays of eight SiP modulators and single-mode fibres. The module offers an aggregate line rate of 448 Gbit/s over distances that are typically found in data-centre and campus-area networks. In a second module, we combine four InP lasers with silicon-based IQ modulators, demonstrating energy-efficient coherent data transmission at an aggregate line rate of 784 Gbit/s over a distance of 75 km. To the best of our knowledge, this represents the highest data rate thus far demonstrated by a SiP transmitter module with hybrid integrated lasers while maintaining a per-channel footprint comparable to that of monolithically integrated InP-based systems. While our demonstrations were focused on transmitter modules for high-speed optical telecommunications, the technology may unlock a wide variety of novel applications that benefit from the advantages of hybrid photonic integration.

## Materials and methods

### Fabrication

All PWB structures were fabricated using a modified commercial two-photon lithography system (Nanoscribe, Photonic Professional GT), equipped with a 40× microscope objective lens (numerical aperture 1.4, field number 25 mm, write field diameter > 500 µm) as well as galvanometer mirrors for rapid beam movement in the lateral directions. Note that PWBs usually fit completely into the accessible write field area. For larger structures, stitch-less lithography based on galvanometric mirrors that are synchronized to sample stage movement could be beneficial^36^. As a lithography light source, we use a fs-laser with a pulse length of 100 fs (FemtoFibre pro NIR, Toptica) and a repetition rate of 80 MHz. The lithography system is equipped with proprietary control software that allows for precise localization of coupling interfaces as well as for automated PWB fabrication with high shape fidelity. In the lithography process, coarse localization of the chips is typically accomplished by marker detection based on a calibrated bright-field image of the system. Additionally, the system is equipped with a confocal imaging unit using the lithography laser and its beam deflectors for the acquisition of 3D images that are perfectly aligned to the lithography coordinate system and hence to any lithographically fabricated structures. For confocal imaging, the laser power is reduced to avoid any unwanted polymerization.

In our current experiments, we use standard writing techniques without taking any measures for process acceleration, leading to fabrication times of typically 30 s … 5 min per PWB, depending on the PWB volume. Currently, the exposure time is dominated by the settling time (100 ms) of the piezo-electric actuator that is used for axial movement of the objective between exposure layers, as well as by the exposure speed. By using the full capacity of current high-speed galvanometer scanners (5000 lines/s for a line length of 40 µm) as well as continuous movement of the piezo-electric actuator, writing times well below 30 s seem reasonable even for large PWB connections.

In the lithography process, the liquid negative-tone photoresist (Nanoscribe IP-Dip, refractive index *n* = 1.52 at 780 nm; see also ref. ^37^) simultaneously acts as an immersion medium for the objective lens. Unexposed photoresist is removed in a two-step development process using propylene-glycol-methyl-ether-acetate (PGMEA) as a developer for 20 min, followed by rinsing in isopropyl alcohol (2-propanol).

### Low-refractive-index cladding

In most cases, a low-refractive-index liquid (Cargille Laser Liquid 3421; *n* = 1.30) is drop-cast onto the assembly to emulate a the waveguide cladding. For more permanent structures, the low-refractive-index liquid can be replaced with a low-index long-term-stable protective coating. The on-chip PWB connections exposed to climate-chamber tests were encapsulated by a low-refractive-index (*n* = 1.36) adhesive.

### Trajectory planning of the PWBs

Each PWB needs to be precisely adapted to the position and the emission direction of the optical coupling interfaces that are to be connected. This requires on-the-fly generation of the corresponding 3D PWB geometry during the fabrication process—another key functionality of our software. In the first step of the PWB design, we calculate a trajectory that is optimized for low curvature and hence low radiation loss. Along this trajectory, the waveguide cross section is extruded to form a 3D model for subsequent fabrication.

### SiP-to-PWB interface

The performance of photonic MCMs crucially relies on broadband and efficient coupling between silicon strip waveguides and 3D freeform PWBs. For coupling to SiP circuits, we use down-tapered silicon waveguide cores that are embedded into up-tapered PWB waveguides; see ref. ^17^ for details. We detect the location of the Si taper by camera-based identification in combination with a local 3D confocal scan.

In our experiments, the SiP chips for the 8-channel IM/DD and the four-channel coherent transmitter originate from the same wafer. To provide direct access to the waveguides for PWB coupling and to enable over-cladding of the SOH slot-waveguide, the top-oxide layer covering the photonic devices must be removed. For the transceiver chips, over-etching occurred during the oxide opening due to a non-optimized process. This led to partial damage of the silicon tapers with tip widths of ~200 nm rather than the designed 130 nm and hence caused increased insertion losses of these PWB interfaces. Note that the passive test chip used for our reliability experiment was not subject to such defects; see Fig. 2. Using defect-free tips, we recently demonstrated^17^ PWB connections of HCSEL to passive SiP chips with losses down to 0.4 dB.

### Fibre-to-PWB interface

Photonic wire bonds were connected to standard single-mode fibres (Corning SMF-28) having a mode-field diameter of (10.3 ± 0.4) µm, defined as the diameter where the intensity has decreased by a factor of 1/e² compared to its maximum value. Details for fibre-chip interfaces can be found in ref. ^15^.

### IM/DD transmitter module

For characterization of the IM/DD transmitter, an arbitrary-waveform generator (AWG, Keysight M8196A) is used to provide the bipolar data signals for the two arms of the MZM. The signals are coupled to the chip using a microwave probe in a signal-ground-ground-signal (SGGS) configuration. A 50 Ω resistor terminates the transmission lines. We bias the MZM at the quadrature point for modulating the light intensity either with on-off-keying (OOK) or with four-level pulse amplitude modulated (PAM-4) signals. We apply pre-equalization to compensate for the AWG frequency response. For the transmission experiments based on PAM-4 signalling, we use pulses with cosine shapes in the time domain. The length of the pseudo-random bit sequence (PRBS) amounts to 2^15^−1. As a receiver, we use a *p*-*i*-*n* photodiode with an integrated transimpedance amplifier (Finisar XPRV2022A-VF-FP). The optical signal is sent either directly to the receiver (back-to-back, B2B), through a 2 km-long SMF (Siecor 1528, attenuation *a*_dB_ = 0.25 dB/km, dispersion coefficient D = 18.5 ps/(nm km) at 1550 nm), or through a 10 km-long SMF (Corning SMF-28, *a*_dB_ = 0.18 dB/km, D = 18 ps/(nm km) at 1550 nm). A high-speed oscilloscope (Keysight DSO-X 93204 A, 80 GSa/s) is used to record the received signals for offline analysis. The received waveforms were analysed by signal processing routines implemented in Python, which comprise filtering, clock recovery, equalization and resampling.

In the experiment, we measure the launch powers at the respective SMF outputs by adjusting the MZMs to full transmission. For Channel 6 of the IM/DD transmitter, the launch power is reduced by an additional 3 dB multi-mode interference coupler (MMI) coupler on the silicon chip. To estimate the losses of the PWB connections, we use the total output power *P*_las_ of the HCSEL prior to photonic wire bonding as a reference and compare it to the power *P*_out_ at the output connector of the MCM. With *P*_las_, *P*_out_, and the measured on-chip device loss of the MZM of typically 5 dB, we estimate the compound insertion loss for the pair of cascaded PWBs in each channel, leading to an average value of 3.6 dB per PWB in the best case and to 5.3 dB per PWB for the case in which one of the structures was affected by a residual dirt particle. This problem, however, is not fundamental and should disappear if all fabrication steps can be performed under clean-room conditions. For the demonstration of the IM/DD transmitter module, the low-index over-cladding was emulated by an index-matching liquid (Cargille Laser Liquid 3421).

### Coherent transmitter module

The losses of the PWB interfaces are estimated by measuring the power levels *P*_launch_ at the output SMF and by comparing them to the emission power *P*_las_ of the HCSEL prior to photonic wire bonding, as well as to the power coupled out of additional on-chip taps (not drawn in Fig. 4c) that are connected to grating couplers; see Supplementary Section S3 for details. For the best PWB connecting the HCSEL to the SiP chip, we estimate a loss of 4.0 dB, and the lowest PWB loss on the fibre side amounts to 5.5 dB. These relatively high losses are mainly caused by the fact that the PWBs are operated without a protective top cladding or index-matching liquid that would reduce the index contrast and hence allow for single-mode operation of the PWBs. Moreover, the efficiency of the coupling interface to the SiP waveguides is impaired by the non-optimum etching of the top-oxide openings applied to the wafer from which both SiP chips used for the system experiments originate; see the discussion of the SiP-to-PWB interface above. Details on the loss estimation technique can be found in Supplementary Section S3.

For characterization of the coherent transmitter, we again use an AWG (Keysight M8196A) to generate the drive signals for the optical IQ modulators. The signals are derived from pseudo-random binary sequences with length 2^11^−1 and pre-equalized to compensate for the measured frequency response of each modulator. At the receiver, the signals are detected by an optical modulation analyser (OMA, Keysight N4391A) acting as a coherent receiver with a built-in external-cavity laser as a local oscillator (LO). The output of the coherent receiver is digitized by a two-channel 80 GSa/s real-time oscilloscope (Keysight DSO-X 93204 A) and recorded for offline digital signal processing (DSP), comprising timing recovery, equalization, frequency offset compensation, carrier phase compensation and decoding. The receiver further comprises an erbium-doped fibre amplifier (EDFA) followed by a bandpass filter (full width at half maximum of 0.6 nm) to suppress out-of-band amplified spontaneous emission (ASE) noise.

### SOH modulator post-processing

For the SOH electro-optic modulators, the organic cladding material is deposited onto the slot waveguides after photonic wire bonding; see Fig. 4b. To avoid contact of the organic EO cladding with the PWB, we used a high-precision dispensing technique that allows deposition of traces with less than 20 µm width via a thin glass needle. To induce macroscopic EO activity, the material in the SOH MZM is poled in a one-time process, enabling efficient push-pull operation of the devices by a single drive signal; see ref. ^27^ for details.

## Supplementary information

Supplemental Material 5
