# Supplementary material for: Hybrid multi-chip assembly of optical communication engines by in situ 3D nano-lithography
Source: Light Sci Appl. 2020 Apr 27;9:71. doi: 10.1038/s41377-020-0272-5 (PMC7184737; doi:10.1038/s41377-020-0272-5)
Supplement: Supplementary file 1 — Supplemental Material 5 [file 41377_2020_272_MOESM1_ESM.pdf]

# Hybrid Multi-Chip Assembly of Optical Communication Engines by In-Situ 3D Nano-Lithography

# - Supplementary Information -

**Matthias Blaicher[1,2,*], Muhammad Rodlin Billah[1,2*], Juned Kemal[1], Tobias Hoose[1,2], Pablo Marin-Palomo[1], Andreas Hofmann[3], Yasar Kutuvantavida[1,2], Clemens Kieninger[1,2], Philipp-Immanuel Dietrich[1,2,4], Matthias Lauermann[1,4], Stefan Wolf[1], Ute Troppenz[5], Martin Moehrle[5], Florian Merget[6], Sebastian Skacel[4], Jeremy Witzens[6], Sebastian Randel[1], Wolfgang Freude[1], Christian Koos[1,2,4]**

*[1]Institute of Photonics and Quantum Electronics (IPQ), Karlsruhe Institute of Technology (KIT), Engesserstraße 5, 76131 Karlsruhe, Germany.*
*[2]Institute for Microstructure Technology (IMT), KIT, Hermann-von-Helmholtz-Platz 1, 76344 Eggenstein-Leopoldshafen, Germany.*
*[3]Institute for Automation and Applied Informatics (IAI), KIT, Hermann-von-Helmholtz-Platz 1, 76344 Eggenstein-Leopoldshafen, Germany.*
*[4]Vanguard Automation GmbH, Gablonzer Straße 10, 76185 Karlsruhe, Germany*
*[5]Fraunhofer Institute for Telecommunications, Heinrich Hertz Institute (HHI), Einsteinufer 37, 10587 Berlin, Germany*
*[6]Institute of Integrated Photonics (IPH), RWTH Aachen, Sommerfeldstraße 18/24, 52074 Aachen, Germany*
**) both authors contributed equally to this work*

## S1 Scalability and stability of photonic wire bonds

For demonstrating environmental stability of photonic wire bonds, five samples were subjected to climate tests. Each chip consists of three independent fields, each field containing 33 PWB bridges, see Fig. S1(a) and Fig. 2 of the main manuscript. The cladding is locally deposited in each field. All five samples were tested at +85°C and 85% relative humidity according to the standard Telcordia test conditions[1]. One of the samples was additionally exposed to 500 cycles of -40°C/+85°C transitions at an average heating/cooling rate of 3 K/min, limited by the performance of our climate chamber. None of these samples showed any sign of fatigue, such as delamination of the cladding material from the SiP chip, or change in colour, and the PWB transmission remained unchanged. Figure S1(b) shows the results of a long-term damp-heat test at 85°C and 85 % relative humidity that was performed with an older sample, containing more than 50 PWB test structures. In this sample, the average insertion loss is slightly higher and amounts to approximately 2 dB, which remain stable over the whole 3500 h of damp-heat test.

We further tested the power handling capabilities of PWB structures by subjecting them to high-power continuous laser radiation at 1550 nm. We observe that the SiP waveguides were destroyed by non-linear absorption at approximately 19 dBm of on-chip power before any damage was observed at the PWB bridge, see Fig. S2.

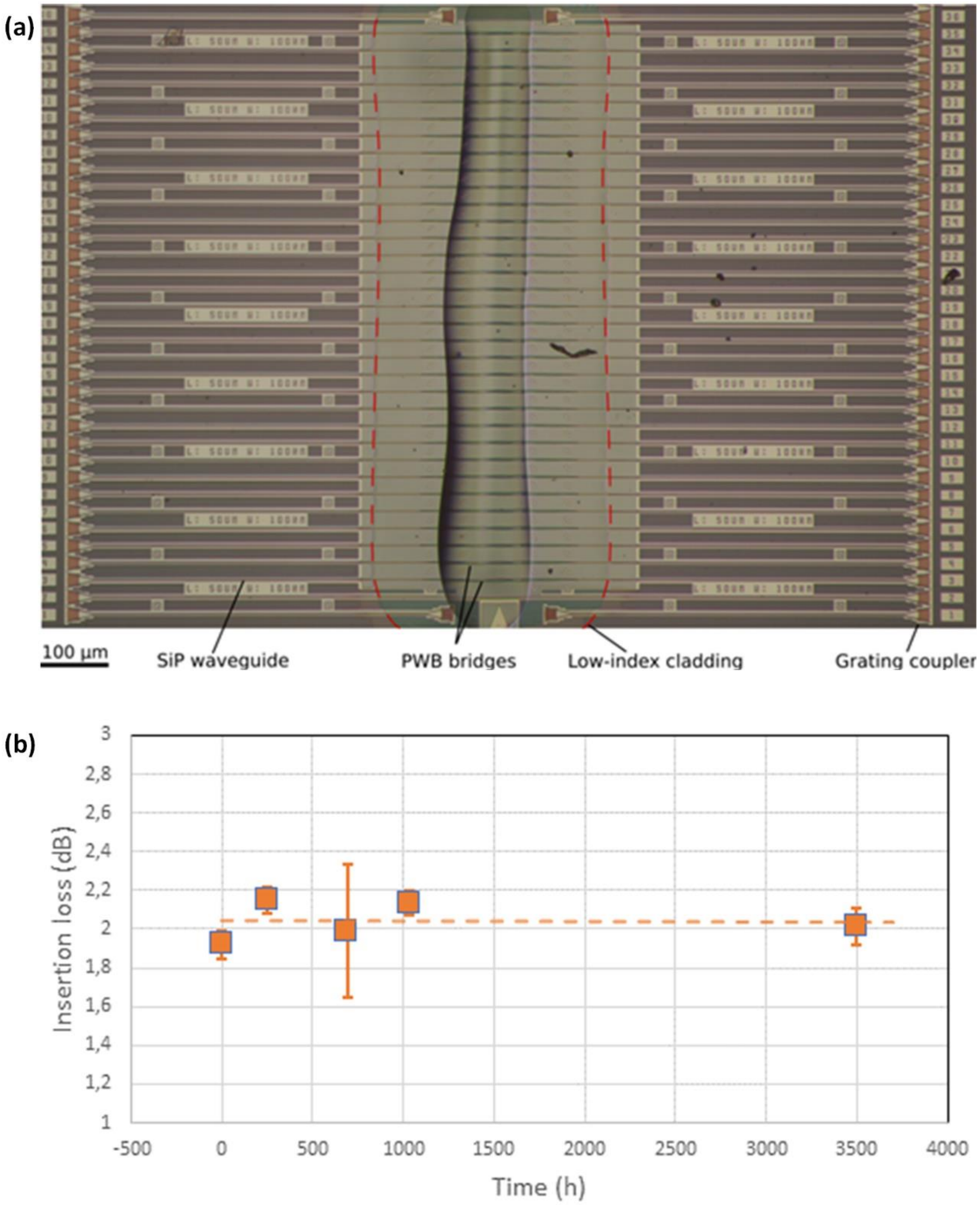


**Figure S1: Scalability and stability of photonic wire bonds. (a)** Micrograph of a field of densely spaced on-chip PWB bridges connecting down-tapered ends of SiP strip waveguides. The PWB are covered with a protective low-index cladding. Fig. 2a of the main manuscript shows bare PWB bridge structures prior to applying the cladding material. The sample has been subjected to temperature 500 cycles of -40°C/+85°C in addition to 500 hours of damp heat test at +85°C and 85% relative humidity. No change in transmission nor any physical changes such as delamination of the cladding material from the SiP chip was observed. **(b)** Long-term damp-heat test of PWB at 85°C and 85 % relative humidity. In this sample, the average insertion loss amounts to approximately 2 dB - slightly higher than in the one shown in Fig. 2 of the main manuscript. This loss remains stable over the whole 3500 h of damp-heat tests.

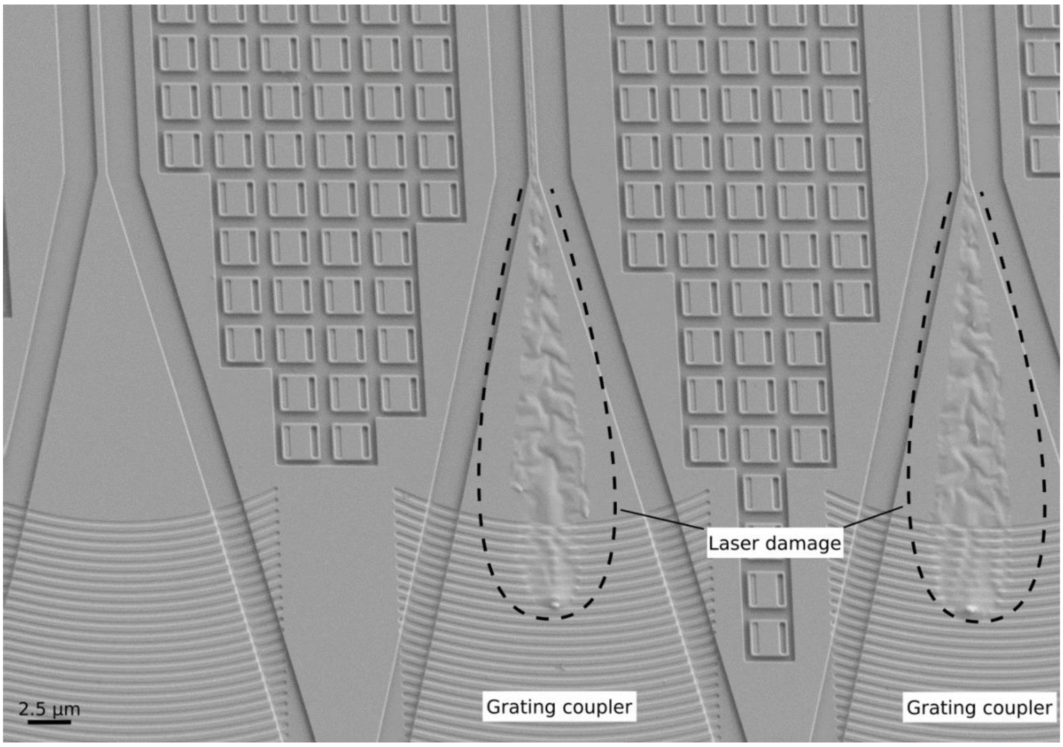


**Figure S2: High-power testing.** In the experiments, we tested the power handling capabilities of structures similar to the ones shown in Supplementary Fig. S1. To this end, CW light at a wavelength of 1550 nm was launched to the input grating coupler (GC) from a standard SMF. The depicted damages occurred at launch powers of approximately 24 dBm in the SMF, which would correspond to 19 dBm of on-chip power taking into account a GC loss of 5 dB. In all experiments, the input GC was destroyed before any damages to the PWB bridges were observed.

# S2 Demonstration 1: Eight-channel multi-chip transmitter module for intensity modulation and direct detection

## S2.1 Electrical channel cross-talk for intensity modulated eight-channel transmitter

In the experiments described in the main text, the various channels were operated one by one due to the lack of an RF package and an adequate number of drive channels. To confirm that simultaneous operation of all channels is possible, we measure the electrical crosstalk between two neighbouring unterminated modulators using a vector network analyser (VNA), see Fig. S3. To this end, we apply a small-signal modulation to one of the RF input ports of the Mach-Zehnder modulator (MZM) belonging to Channel 7 (Ch 7), and we measure the cross-talk signals at the ends of the transmission lines of the neighbouring MZM belonging to Ch 8 and Ch 6, see green and red trace in Fig. S3. In addition, we determine the direct transmission through the MZM transmission lines of Ch 7 and Ch 8 in a separate measurement, see blue and orange trace in Fig. S3. We obtain an electrical crosstalk of approximately -20 dB or less for the relevant frequency range below 40 GHz. Note that this measurement corresponds to a conservative estimate in the sense that the MZM transmission line of Ch 7 was left unterminated while measuring the cross-talk to the adjacent channels. We expect that proper termination of all MZM transmission lines would further decrease the crosstalk. These experiments indicate that parallel operation on all channels should be possible once dedicated RF boards and driver electronics are available.

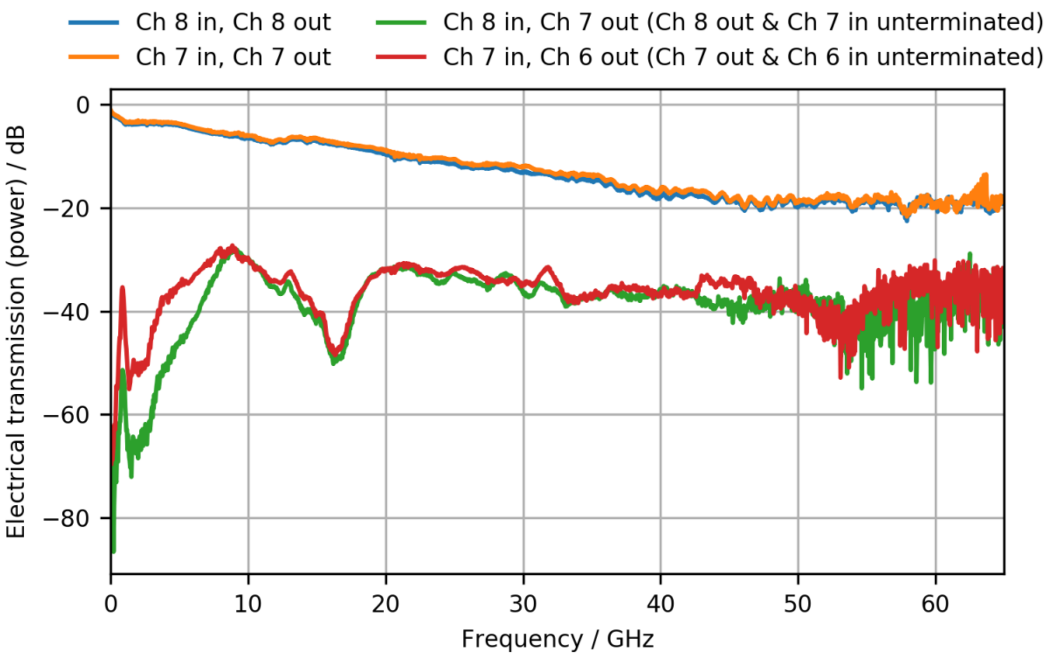


**Figure S3: Electrical cross-talk of MZM:** We measured the cross-talk from the unterminated Channel 2 (Ch 2) to adjacent channels (green and red traces), along with the RF transmission through the MZM transmission lines of Ch 1 and 2 (blue and orange trace).

## S2.2 Bit-error ratio (BER) estimation of a PAM-4 intensity-modulated signal

Given the limited length of our recordings, we estimate the bit error ratio (BER) based on the measured probability density functions (PDF) at the optimal sampling point. Such approaches are well established for on-off-keying (OOK) signals[2], and models exist for PAM4-signalling[3]. In the following, we extend the existing models by introducing independent Gaussian probability density functions $w_i(u)$ for each level of index $i$ with a mean of $u_i$ and standard deviation of $\sigma_i$. In our experiments, the assumption of Gaussian distributions is justified since we did not use any optical amplifiers such that the noise originates predominantly from the electric amplifier at the receiver. In the following, we denote the probability of transmitting a one or a zero with $p(1\mathrm{t})$ or $p(0\mathrm{t})$, respectively. The optimum decision threshold $u_{\mathrm{th}}$ for a minimum BER is found by calculating a stationary point of the BER with respect to the decision threshold, i.e., $\mathrm{d}\,BER/\mathrm{d}\,u_{\mathrm{th}} = 0$, where

$$\mathrm{BER} = p(1\mathrm{t})\int_{-\infty}^{u_{\mathrm{th}}} w_1(u)\,\mathrm{d}u + p(0\mathrm{t})\int_{u_{\mathrm{th}}}^{+\infty} w_0(u)\,\mathrm{d}u\,, \qquad p(1\mathrm{t})w_1\big(u_{\mathrm{th,opt}}\big) = p(0\mathrm{t})w_0\big(u_{\mathrm{th,opt}}\big).$$

In case of OOK signals, the optimal decision threshold $u_{\mathrm{th}}$ is often calculated[2] based on the assumption that the ratio of occurrence probabilities of ones and zeros is equal to the ratio of the corresponding standard deviations, i.e., $p(1\mathrm{t})/p(0\mathrm{t}) = \sigma_1/\sigma_0$. In this case, the optimum decision threshold is found to be $u_{\mathrm{th,opt},\sigma} = (u_0\sigma_1 + u_1\sigma_0)/(\sigma_0 + \sigma_1)$. In our experiments we do not adjust the transmit probabilities of ones and zeros to the associated standard deviations, but rather transmit all symbols with equal probability. The optimal decision threshold $u_{\mathrm{th,opt}}$ and the resulting BER are then given by

$$u_{\mathrm{th,opt}} = \frac{\mu_1\sigma_0^2 - \mu_0\sigma_1^2 - \sigma_1\sigma_0\sqrt{\mu_0^2 - 2\mu_1\mu_0 + \mu_1^2 + \log\left(\sigma_0^{2\sigma_0^2-2\sigma_1^2}\sigma_1^{2\sigma_1^2-2\sigma_0^2}\right)}}{\sigma_0^2 - \sigma_1^2}$$

$$\mathrm{BER} = -\frac{1}{4}\mathrm{erf}\left(\frac{\sqrt{2}\mu_0\sigma_1 - \mu_1\sigma_1 + \sigma_0\sqrt{\mu_0^2 - 2\mu_1\mu_0 + \mu_1^2 + \log(\sigma_0^{2\sigma_0^2-2\sigma_1^2}\sigma_1^{2\sigma_1^2-2\sigma_0^2})}}{2\sigma_0^2 - 2\sigma_1^2}\right) + \frac{1}{4}\mathrm{erf}\left(\frac{\sqrt{2}\mu_0\sigma_0 - \mu_1\sigma_0 + \sigma_1\sqrt{\mu_0^2 - 2\mu_1\mu_0 + \mu_1^2 + \log(\sigma_0^{2\sigma_0^2-2\sigma_1^2}\sigma_1^{2\sigma_1^2-2\sigma_0^2})}}{2\sigma_0^2 - 2\sigma_1^2}\right) + \frac{1}{2}$$

In case of PAM4, the four signal levels $u_i$, $i = 1 \ldots 4$, can analogously be described by four Gaussian probability density functions. Similarly to OOK, we choose the optimal decision $u_{\mathrm{th,opt},i,j}$ threshold between two adjacent levels $u_i$ and $u_j$ by considering the associated Gaussian distributions of the two adjacent levels similarly to the procedure described in above. Note that this approach assumes negligible contributions of the Gaussian probability density functions that are associated with additional signal levels $u_k$ that are not adjacent to the considered decision threshold, i.e., for which $k \neq i$ and $k \neq j$. However, when calculating to the overall BER, we do include the contribution of erroneously detected symbols that correspond to non-adjacent signal levels, i.e., we take into account the fact that large deviations from the transmitted signal level may result in more than one bit error per symbol. Since the full expression for the PAM-4 bit-error ratio $BER_{\mathrm{PAM4}}$ is too long for print, we provide a Python-based computer algebra derivation as a separate Supplementary Listing 1. The program allows analyzing intensity modulation with an arbitrary number of levels for arbitrary bit-to-level mapping and symbol probabilities.

## S2.3 Data transmission results

For OOK encoded signals, no errors were recorded for all data rates and transmission distances irrespective of the use of post-equalization. The BER could hence only be estimated. For the estimation, we consider the post-equalized signals, since the non-equalized signals suffer from inter-symbol interference (ISI), which renders the assumption of additive white Gaussian noise invalid. For PAM-4 encoded signals, our recordings included errors for high symbol rates and longer distances, as shown in Fig. S4. Note that reliable measurement of the BER requires at least 13 bit errors[2], resulting in an upper boundary of $\mathrm{BER}_{\mathrm{measured}} \gtrsim 10^{-4}$ for our recordings, which is indicated as a grey bar in Fig. S4. All estimated and directly measured BER are below the of $3.8\times10^{-3}$ limit of hard-decision forward-error correction (HD-FEC) with 7 % coding overhead[4,5]. Note that the estimated and the measured BER coincide pretty well for the cases where direct BER measurements were possible, indicating the validity of the approach.

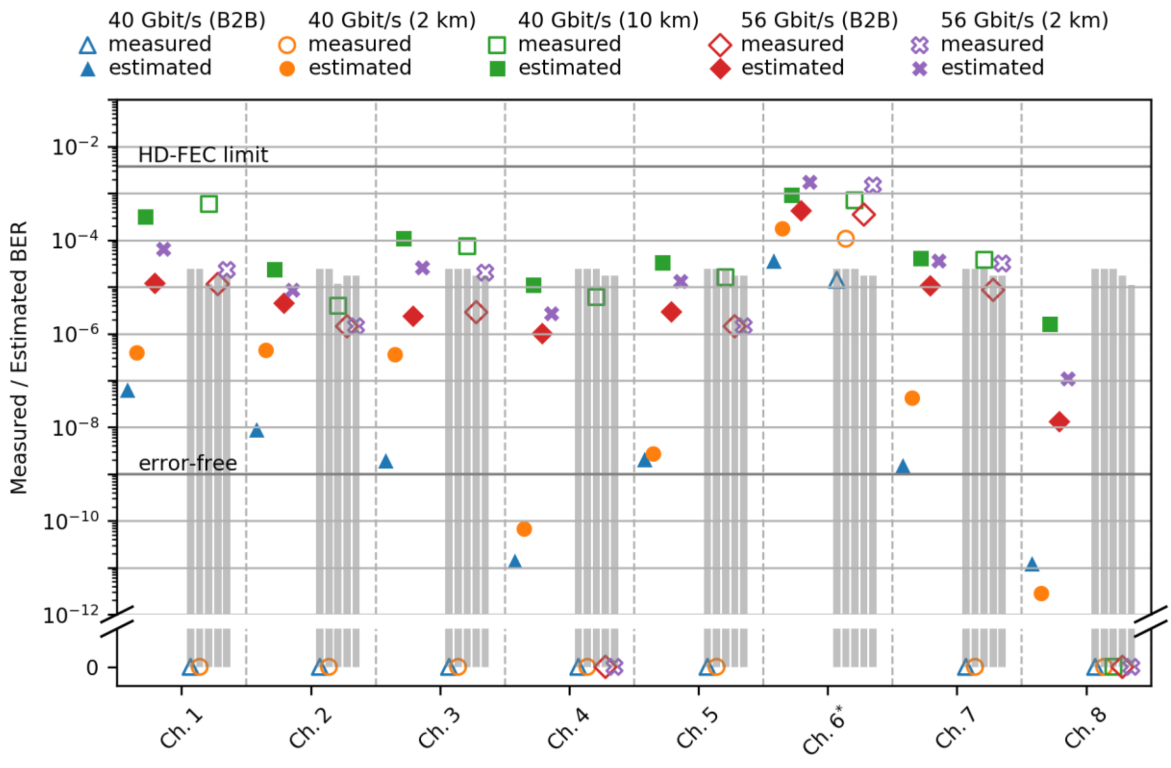


**Figure S4: Measured (open symbols) and estimated (filled symbols) bit error ratios (BER) of the eight-channel multi-chip module transmitting PAM-4 signals at different symbol rates and over different distances.** For all transmission demonstrations, the BER stays below the 7% HD-FEC threshold, including Channel 6, which suffers from additional losses due to an avoidable extra on-chip MMI. The aggregate line rate of the module amounts to 448 Gbit/s. For each transmission demonstration, the grey bars indicate the BER range for which the measured BER is not statistically relevant since less than 13 few errors were detected within the limited recording length which varied between $4.94 \cdot 10^{5}$ and $1.08 \cdot 10^{6}$ bits. Measured BER values (open symbols) at the very bottom ('BER = 0') indicate measured recordings which did not contain any error.

## S3 Demonstration 2: Four-channel multi-chip transmitter module for coherent communications

The insertion losses (IL) of the PBW connections are obtained by measuring the power levels at various sampling points and by taking into account the grating coupler (GC) losses as well as the losses of the various on-chip components. The GC and component losses were derived from measurements of nominally identical reference structures produced in the same fabrication run as the transceiver chips. Specifically, we measure the power levels $P_{\text{launch}}$ at the output SMF of each channel and compare them to the emission power $P_{\text{las}}$ of the corresponding HCSEL prior to photonic wire bonding. In addition, we measure the power coupled out of additional on-chip taps at the output of the MZM of each channel. To obtain the emission power $P_{\text{las}}$ of each HCSEL, we characterize the devices as described in Ref. [6] prior to photonic wire bonding, leading to power levels of 7.4, 8.4, 8.6, 9.0 dBm for Channel 1, 2, 3, and 4 and a drive current of 100 mA, respectively. The corresponding fiber-coupled module output powers $P_{\text{launch}}$ amount to -19.9 dBm, -13.6 dBm, -12.9 dBm, and -11.6 dBm, respectively, all measured for maximum transmission of the SOH in-phase/quadrature (IQ) modulators. In this case, the on-chip loss of the SOH IQ amounts to (8.0 ± 0.3) dB, including bus waveguide losses of the order of 1.5 dB. The remaining losses are attributed to non-ideal power splitters, strip-to-slot transitions in the SOH MZM, and imperfectly etched slot waveguides. Based on these measurements, we estimate HCSEL-to-SOI PWB losses of 10.8 dB, 4.9 dB, 4.0 dB, and 6.9 dB for Channel 1, 2, 3, and 4, respectively. The high loss of Channel 1 is caused by a dirt particle sticking to the PWB. The chip-to-fibre PWB losses amount to 8.8 dB, 9.5 dB, 9.2 dB, and 5.5 dB, respectively. Note that the module output power used in the transmission experiment is smaller than the maximum achievable launch power $P_{\text{launch}}$ due to the modulation loss

of the individual MZM and due to the intrinsic 3 dB loss that occurs at the I/Q power combiner when adjusting for an ideal π/2 phase difference.

For simplicity, Fig. 4(d) of the main manuscript only shows the results for 28 GBd and 56 GBd transmission over 75 km. We also performed measurements at 45 GBd as well as back-to-back reference experiments. The results are given in Fig. S5.

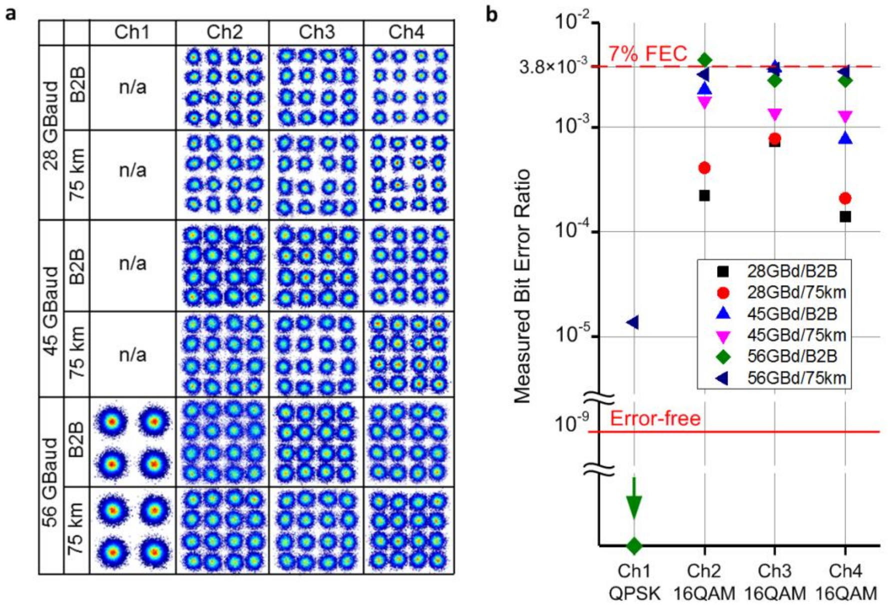


**Figure S5: Coherent transmission experiments, including back-to-back measurements as well as transmission at 45 GBd. (a)** Constellation diagrams for transmission of all channels at 28 GBd, 45 GBd, and 56 GBd. The performance of Ch1 was impeded by a dirt particle on one of the PWB, leading to a lower launch power, such that only QPSK transmission was successful. For simplicity, we only confirmed error-free transmission performance at the highest symbol rate and did not perform any experiments at 28 Gbd or 45 GBd. **(b)** Measured bit error ratios (BER) for all channels. Channel 1 (Ch 1) was operated with QPSK only, whereas Ch 2 … 4 are tested with 16QAM signals. All BER values stay below the threshold for 7% FEC except for the 56 GBd back-to-back operation (B2B) of Ch 2. Since the BER of the 75-km transmission is below the FEC limit, we attribute this result to a non-optimum adjustment of the modulator bias in the B2B experiment. The aggregate module line rate amounts to 784 Gbit/s.

## S4 References


1. Telcordia. GR-468-CORE: Reliability Assurance for Optoelectronic Devices. (2004).

2. Freude, W. *et al.* Quality metrics for optical signals: Eye diagram, Q-factor, OSNR, EVM and BER. in *2012 14th International Conference on Transparent Optical Networks (ICTON)* **1**, 1–4 (IEEE, 2012).

3. Proakis, J. *Digital Communications*. (McGrawHill, 2000).

4. Chang, F., Onohara, K. & Mizuochi, T. Forward error correction for 100 G transport networks. *IEEE Commun. Mag.* **48**, 48–55 (2010).

5. International Telecommunication Union - ITU-T. Forward error correction for high bit-rate DWDM submarine systems. *Series G.975.1* 1–58 (2005).

6. Billah, M. R. *et al.* Hybrid integration of silicon photonics circuits and InP lasers by photonic wire bonding. *Optica* **5**, 876 (2018).
